# LoWMob: Intra-PAN Mobility Support Schemes for 6LoWPAN

**DOI:** 10.3390/s90705844

**Published:** 2009-07-23

**Authors:** Gargi Bag, Muhammad Taqi Raza, Ki-Hyung Kim, Seung-Wha Yoo

**Affiliations:** 1 Information and Communication Department/Ajou University, South Korea; E-Mails: kkim86@ajou.ac.kr (K.-H.K.); swyoo@ajou.ac.kr (S.-W.Y.); 2 USN Networking Research Team / Electronics and Telecommunications Research Institute (ETRI), Korea; E-Mail: taqi@etri.re.kr

**Keywords:** sensor networks, 6LoWPAN, network-based mobility support

## Abstract

Mobility in 6LoWPAN (IPv6 over Low Power Personal Area Networks) is being utilized in realizing many applications where sensor nodes, while moving, sense and transmit the gathered data to a monitoring server. By employing IEEE802.15.4 as a baseline for the link layer technology, 6LoWPAN implies low data rate and low power consumption with periodic sleep and wakeups for sensor nodes, without requiring them to incorporate complex hardware. Also enabling sensor nodes with IPv6 ensures that the sensor data can be accessed anytime and anywhere from the world. Several existing mobility-related schemes like HMIPv6, MIPv6, HAWAII, and Cellular IP require active participation of mobile nodes in the mobility signaling, thus leading to the mobility-related changes in the protocol stack of mobile nodes. In this paper, we present LoWMob, which is a network-based mobility scheme for mobile 6LoWPAN nodes in which the mobility of 6LoWPAN nodes is handled at the network-side. LoWMob ensures multi-hop communication between gateways and mobile nodes with the help of the static nodes within a 6LoWPAN. In order to reduce the signaling overhead of static nodes for supporting mobile nodes, LoWMob proposes a mobility support packet format at the adaptation layer of 6LoWPAN. Also we present a distributed version of LoWMob, named as DLoWMob (or Distributed LoWMob), which employs Mobility Support Points (MSPs) to distribute the traffic concentration at the gateways and to optimize the multi-hop routing path between source and destination nodes in a 6LoWPAN. Moreover, we have also discussed the security considerations for our proposed mobility schemes. The performance of our proposed schemes is evaluated in terms of mobility signaling costs, end-to-end delay, and packet success ratio.

## 1. Introduction

The IEEE 802.15.4 standard [1] has emerged as a strong technology for Wireless Sensor Networks (WSNs) to morph Personal Area Networks (PANs) into Low power Wireless Personal Area Networks (LoWPANs). LoWPANs are characterized by low data rates, low power consumption, low costs, autonomous operations, and flexible topologies. In order to fully realize a pervasive or ubiquitous environment, LoWPANs must be connected to the Internet. Internet Engineering Task Force (IETF) is standardizing the transmission of IPv6 over LoWPANs through 6lowpan (IPv6 over Low power Wireless Personal Area Networks) working group [2]. The emerging application range of 6LoWPAN includes consumer appliances, home automation, monitoring and control in industrial environments, military, and environmental monitoring, etc.

Incorporating mobility in 6LoWPAN [3] can lead to the realization of new and exciting applications. For example, mobility of 6LoWPAN devices can be exploited in health care applications. In this case, the patients have sensor nodes embedded in their clothes for sensing some of their important health parameters like pulse rate and temperature, etc. These sensor nodes can sense the data and transmit it to a monitoring facility, even when the patient is moving, by using one of the existing communication technologies like WLAN and UWB. However, these technologies are more applicable when the application requires high data rate and often demand complex link and physical layer solutions with complicated hardware. 6LoWPAN has been considered as the most suitable technology for supporting mobility in sensor networks due to their low power and low data rate characteristics. Also, 6LoWPAN enables the integration of IEEE802.15.4 networks with the Internet, thereby increasing the monitoring scope of the patient.

In order to prevent packet losses due to mobility, some of the existing tunnel-based mobility protocols like HMIPv6 [4], FMIPv6 [5], and MIPv6 [5] can be used. These schemes can help a Mobile Node (MN) to maintain its ongoing communication with the outer world and to minimize the packet losses while it is moving. However, the above mentioned schemes are host-based mobility protocols in which the MN actively participates in mobility-related signaling. Moreover, these are network layer solutions that provide mobility-related features at the IP layer. In other words, mobility-related packets are carried by the IP traffic [6]. Similarly, routing-based mobility management schemes like HAWAII [7] and Cellular IP [5] require the MN to manage its mobility by sending *path-setup* messages and periodic *path-refresh* messages [7]. Also, in HAWAII, the domain router could become a potential bottleneck, as all of the MN’s packets are routed through it. Another mobility support protocol is NEMO that requires a mobile router (MR) to support the mobility of a PAN [8].

PMIPv6 [9] is a network-based mobility support protocol currently being standardized by IETF’s netlmm (Network-based localized mobility management protocol) working group. PMIPv6 could be considered as a suitable candidate to enable mobility in 6LoWPAN devices, as in the scheme the network handles all the mobility-related signaling on behalf of MNs. However, at its current phase, PMIPv6 defines the interface between the Mobile Access Gateway (MAG) and a MN for one-hop communication at the network layer. It does not specify the interface between MN and MAG for multi-hop communication. Also the mobility-related packets exchanged between network entities are carried by IP traffic. Moreover, PMIPv6 demands another level of tunneling overhead at the network layer between the Gateway (GW) and the MAG that serves MNs.

This paper proposes a local mobility support scheme for a mobile 6LoWPAN node at the adaptation layer of 6LoWPAN. In other words, the mobility-related packets are delivered at the adaptation layer of 6LoWPAN. The proposed mobility support scheme is network-based and provides multi-hop communication between the GW and the MN via Static Nodes (SNs) (Note that in this paper, by Static Nodes we mean the static sensor nodes of the network (PAN); we named so in order to avoid any ambiguity between the MN (which is also a sensor node) and the static sensor nodes of the network.). Thus our schemes allow the MNs to communicate with the GW through short range IEEE 802.15.4 communication links that help the MNs to conserve power and extend the lifetime of the overall networks including MNs. Also the proposed scheme takes into consideration the sleep state of the SN.

The paper further presents a distributed version of the LoWMob, named as the Distributed LoWMob (DLoWMob) scheme. The aim of DLoWMob is two folds; firstly it tries to reduce the number of Location Update (LU) messages to the GW by introducing the concept of Mobility Support Points (MSPs) which could extend the overall lifetime of the network. MSP acts as a master node for a set of SNs and handles all the mobility related functionality. Secondly, it supports route optimization between source and destination MNs in a PAN, so that the data packets are not routed via GW.

Moreover, we have also discussed the security considerations for our proposed mobility schemes. The main objective of proposed security mechanism in this paper is to provide a security solution to our proposed mobility schemes.

The performance evaluation compares the end-to-end delay and packet success ratio between source and destination MN in a PAN at different speed of MN in LoWMob, DLoWMob, and DLoWMob with Route Optimization scheme. The handoff signaling costs for the LoWMob and DLoWMob schemes are determined through analytical modeling.

The remainder of the paper is organized as follows. Section 2 describes the related work followed by the 6LoWPAN mobility requirements in Section 3. Section 4 defines the system model and assumptions. Section 5 and 6 describe LoWMob and DLoWMob respectively in detail. Security considerations for our proposed schemes are discussed in Section 7. Performance evaluation follows next in Section 8, and finally Section 9 concludes the paper.

## 2. Related Work

Mobility of 6LoWPAN can give rise to new and exciting applications. One of the possible applications that exploit mobile sensor nodes is healthcare, where patients wear multiple sensors so that their important health parameters like pulse rate, heart beat etc, would be monitored while they are moving. These sensor nodes form a Wireless Body Sensor Network (WBSN) and use mesh routing in order to transmit their readings to a hub or controller, which can be a PDA or a Laptop. Several existing communication technologies have been considered as a candidate of internal and external communication of WBSN. However, most of these protocols have their own shortcomings when they are used in WBSN. For example, WLAN technology is not suitable for WBSN as the low powered WBSN devices have to increase their transmission power in order to avoid interference from other powerful devices like PDA and notebook. Similarly, some communication technologies like Ultra Wideband (UWB) need complex protocols and hardware which may not be feasible for WSBN. Authors in [10] have pointed out that 6LoWPAN can be one of the most suitable technology for WBSN since it is based on the IEEE 802.15.4 specifications. The IEEE 802.15.4 standard implies low data rate, low power, more sleeping time and less complex protocols and hardware for the sensor nodes. Its interaction with IPv6 implies that the sensor nodes should easily be interoperable with all other IP networks, including the Internet. This means that the sensor data can be accessible anywhere from the world.

In [11] the authors have proposed the design of micro mobility support for a sensor node, roaming across several Access Points (AP) of a Bluetooth sensor network. A mechanism to assign IP addresses to an AP and to a sensor node so that a MN would be identified without using the channel number were also proposed. The authors also designed a middleware to carry IP packets over Bluetooth. However, they assume that the sensor node is capable to perform single hop communication even if the MN is not that close to the AP. Moreover, the MNs are expected to incorporate a middleware layer changes in their stack in order to support their mobility.

Another form of mobility is network mobility or NEMO whereby the whole network moves together and their mobility is handled by MR [8]. When the network moves into a new network, the MR acquires a Care of Address (CoA) on its egress interface and sends a LU to its Home Agent (HA). The CoA informs the HA that all of the packets destined to any address derived from MR’s prefix should be forwarded to the MR’s CoA. However, in this scenario, the MR is comparatively more powerful as compared to a single sensor node. Thus special protocols have to be developed in order to handle the mobility of a single mobile sensor node.

The authors in [12] have proposed an interoperable architecture between NEMO and 6LoWPAN in order to support network mobility. They have suggested an extended routing scheme for MRs to support mobility in 6LoWPAN sensor nodes. However, the architecture does not cater for individual node’s mobility.

The existing localized mobility-related protocols can be broadly classified into two groups. These are host-based and network-based protocols. Host-based solutions require MN involvement at the IP layer and software changes at the host’s stack. The host-based mobility protocols like MIPv6, FMIPv6, and HMIPv6 were proposed by IETF [2]. In the network-based mobility protocols, the local IP mobility is handled without involvement of the MN and also the MN does not have to implement a new mechanism in its stack.

In MIPv6, when a MN moves from one network to the other, it forms a CoA based on the prefix of the foreign link. Thereafter, the node (host) itself sends the Binding Update (BU) to it’s HA. When the MN receives a link layer indication of its movement it can solicit a router advertisement. Once the MN knows that IP layer mobility has taken place, it forms a new CoA by using the prefix advertised on the new link and again sends a BU to its HA.

HMIPv6 adds another level on MIPv6 and separates global mobility from local mobility. HMIPv6 was designed so that the MNs can move within a particular domain without having to update their HA or Correspondent Nodes (CNs) when every time they move. It introduces a new entity called Mobile Anchor Point (MAP) which acts as a local HA of the MNs [4]. When a MN moves into a new MAP domain, it configures two CoAs: a Regional CoA (RCoA) on the MAPs link and On-Link CoA (LCoA). It then sends a local BU to the MAP. Following a successful registration with the MAP, a bidirectional tunnel between the MN and the MAP is established. All packets sent by the MN are tunneled to the MAP and vice versa. It should be noted that every time the MN moves within the network, it acquires a new LCoA and sends a local BU to the MAP. However, the RCoA remains unchanged.

FMIPv6 is another enhancement of MIPv6 that aims to reduce the handoff delays for mobile connections. It is designed to allow MNs to anticipate their IP layer mobility. In other words, the MNs can discover the new router prefix before being disconnected from the current router.

However, as discussed before, the host based mobility protocols involve most of the signaling on the MN’s end. This signaling overhead is not appropriate for 6LoWPAN nodes. These protocols are also network layer solutions that provide mobility-related features at the IP layer. Thus mobility-related signaling packets are carried by IP traffic. However, this solution proves to be expensive if they are directly implemented in 6LoWPAN that supports MNs. Moreover, these protocols demand a complex host stack which is not feasible for 6LoWPAN. Additionally, they assume the anchor points of the MN are always available and do not need to go to sleep state. Also, from the security point of view the changes in the temporary local address mean that the MN exposes its topological location to eavesdroppers.

The other host based mobility protocols are HAWAII [7] and Cellular IP [5]. In order to forward MN’s packets, these protocols involve establishment of host specific routes in the routers. The creation of host-specific routes is initiated and updated by the MNs. Thus these protocols require an active participation from the MNs that can negatively impact MN’s lifetime. Also, in HAWAII, when the routers within the domain receive a packet from another MN; then they forward the packets to a router, named as “the Domain Route Router”. This limits the scope of route optimization even if when the corresponding MNs are near to each other.

In the network based localized mobility schemes like PMIPv6 [9], proposed by IETF, the network side performs the mobility-related signaling on behalf of the MN. PMIPv6 introduces several new entities like Localized Mobility Anchors (LMA) and MAG. The mobility entities in the network will track the MN's movements, initiate the mobility signaling and set up the required routing state [9]. However, more specifically, MAG is responsible for tracking the MN's movements to and from the access link and for signaling the MN's LMA [9]. PMIPv6 has several advantages over the host based mobility protocols, thus making it better candidate for 6LoWPAN mobility management. Firstly, PMIPv6 can be compatible with any global mobility management protocols that are not MIPv6, such as Host Identity Protocol (HIP), IKEv2 Mobility, and Multihoming (MOBIKE) [13]. While it is possible that existing localized mobility management protocols could be used with HIP and MOBIKE, some would require additional efforts to implement, deploy, or even in some cases specify in a non-Mobile IPv6 mobile environment [13]. Also, in PMIPv6, the MN does not need to incorporate any changes in the stack. Moreover, since the MN does not configure a LCOA every time it moves within a domain; the handoff latency is minimized as there is no need to perform Duplicate Address Detection (DAD) of the IP address. On the other hand, the interface between the MN and MAG is applied for one- hop communication at the network layer. The interface between the MAG and a MN is not defined for multi-hop communication between GW and MN. Also the mobility-related signaling packets between LMA and MAG are carried by the IP traffic. Moreover, at the network layer, PMIPv6 demands an extra tunneling overhead between the MAG and LMA.

The authors in [14] proposed a network mobility management scheme for wireless mesh networks. The wireless mesh network is composed of several Routing Access Points (RAPs) that not only serve as an access point for the MNs, but also route MN’s data packets. However, the RAPs are much more powerful devices, in terms of processing power and energy consumption, as compared to the 6LoWPAN SNs.

In [15–17], we have introduced mobility management architecture for 6LoWPAN. The architecture considered the mobility of a single 6LoWPAN node within a PAN with multiple GWs and with the help of the existing static nodes. However, the proposed architecture required a SN to send a LU to GW every time a new MN associates with it. This reduced the lifetime of the SNs and eventually the whole PAN. Moreover, the proposed architecture did not consider route optimization between multiple MNs located in the same PAN. This paper aims to reduce the number of LUs sent from SNs to the GW by proposing a distributed version of the architecture presented in [15–17]. The proposed scheme further considers route optimization between multiple corresponding MNs, located in the same PAN.

## 3. Possible Mobility Scenarios in 6LoWPAN

**Figure 1.**
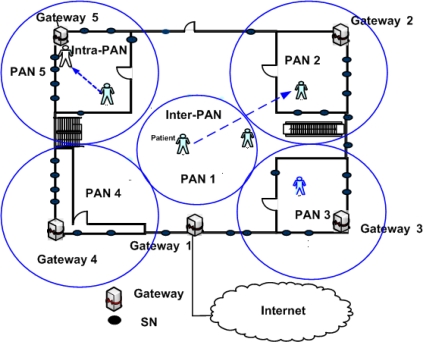
Possible mobility scenarios.

Figure 1 shows a hospital building where each room hosts a PAN. The PANs are comprised of SNs and a GW that connects the PAN to the outer world. A patient can have a sensor node attached, which gathers his/her vital heath parameters like temperature and pulse rate etc. The sensor node then communicates these parameters to the GW, by transmitting the information to its nearest SN. The SN then forwards the information to the GW via other SNs in the PAN. The following lists three possible categories of the mobility types in this scenario:

1. *Intra-PAN mobility*: When a MN moves within a PAN, Intra-PAN mobility occurs. In Figure 1, this kind of mobility occurs when a patient moves within a room.
2. *Inter-PAN mobility:* This kind of mobility occurs when a MN moves from one PAN to another. For example, in Figure 1, a patient moves from one room to the other, where each room hosts one PAN.
3. *WPAN mobility*: In this case, the whole PAN changes its point of attachment. This case is similar to NEMO.

In this paper, we limit our discussion to the Intra-PAN mobility category only. In order to support mobility in the resource constrained devices; the IETF specifies some mobility requirements in 6LoWPAN. These include [3]:

1. Providing fast handoff detection.
2. The MNs should be addressable by any CN, irrespective of its whereabouts.
3. The signaling is also required to be minimized by considering the resource constraint characteristic of the LoWPAN devices.
4. Reduced Function Devices (RFDs) should be kept out of the mobility-related signaling.

## 4. System Model and Assumptions

Our proposed mobility schemes are based on the following network assumptions:

1. A PAN consists of several SNs, which are Full Functional Devices (FFDs) and a PAN coordinator, i.e., the GW. Multiple GWs/PANs can also be considered to improve the reliability and scalability. However, in this paper, we limit our scope for a network having single PAN coordinator.
2. The nodes at the periphery of the PAN are known as Border Nodes (BNs). These are FFDs which are mostly in quasi-sleep state. In quasi-sleep state, the node’s sensor is turned on, whereas its transceiver is turned off.
3. A MN is an FFD or RFD which moves within a PAN. A MN is assumed to transmit or receive data packets periodically, i.e., if a MN does not transmit or receive data packet within MST (Maximum Sleep Time), it is assumed to be dead.
4. The SNs spend most of their time in the sleep state.
5. A MN is an active device which moves within a PAN. It can be a FFD or a RFD.
6. The SNs are densely deployed to an extent that their transmission ranges overlap.
7. Each 6loWPAN device has the same transmission signal strength and receiver sensitivity.
8. Each SN measures the distance between itself and the MN based on the Received Signal Strength Indication (RSSI) [18].
9. Each SN is equipped with a radio-triggered hardware component that activates sensors from/to the sleep state, by sending a special wake up radio signal [19].
10. 6LoWPAN networks provide two types of addresses, IEEE EUI 64-bits extended address, and 16-bits short addresses. The SNs are assigned with IPv6 and 16 bits short address [20].
11. The MN is assigned with a 16 bits short address, which is unique within a PAN, and remains fixed irrespective of its location within the PAN. The IP address of the MN also remains fixed irrespective of its mobility within the PAN.
12. The interoperability between IPv6 domain and the IEEE802.15.4 device is handled by the adaptation layer [20].
13. Each SN is equipped with an antenna array in order to obtain the Angle of Arrival (AoA) measurements.
14. Any of the MAC protocols, synchronous or asynchronous, can be used to ensure the reliability and routing of packets in duty cycle WSN.

## 5. LoWMob Mobility Support Scheme

LoWMob defines the interface for multi-hop communication between the GW and the MN. It thus enables MN’s to utilize short range IEEE802.15.4 links to send their data packets to the GW, even though it is far away from the GW. In this effort, our scheme proposes to utilize SNs to route MNs packets to or from the GW. However, the proposed scheme takes into consideration that SNs themselves are resource constrained devices and periodically switch off to sleep state in order to conserve energy.

Like PMIPv6, LoWMob is a network based mobility scheme where the SNs and the GW are responsible for providing mobility support to the MN. However, in PMIPv6 the mobility-related features are provided at the network layer. In other words, mobility-related signaling packets are carried by IP traffic [6]. LoWMob proposes the mobility support in an adaptation layer of 6LoWPAN.

The LoWMob mobility support scheme can be divided into two parts. The first part deals with the case when a new MN enters the PAN for the first time. The second part discuses the handoff support for MNs when they move within the PAN. The aim of the handoff support is to prevent packet losses. Note that, for our LoWMob scheme, we have only discussed a single MN’s communication scenario with the GW, or with the static CN – that can be within or outside the PAN. Because the purpose of introducing the LoWMob scheme is to introduce the concepts and techniques related to the mobility in 6LoWPAN. Furthermore, we have improved the scheme by proposing a distributed version of it. The distributed version considers route optimization between multiple corresponding MNs in a PAN.

### 5.1. New MN Joining the PAN

**Figure 2.**
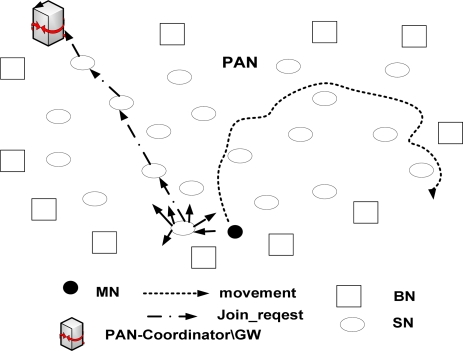
The MN as joining the PAN.

Figure 2 shows a PAN with multiple SNs, a single GW/PAN coordinator and a MN which enters the PAN for the first time. When the BNs detect a movement, they switch from quasi-sleep state to active state. In active state, they frequently transmit beacons. The beacons contain the information about the SN’s short address and PAN ID. Once the MN receives the beacon, it checks the PAN ID and determines whether it has moved to a new PAN or not. If the MN is in different PAN, it sends a *join_request* message to the SN from which it receives the beacon of highest signal strength. The *join_request* message indicates to the SN that the MN is new to the PAN and wants to associate with the PAN. The message contains information regarding MN’s Home address and its EUI 64 bits address. The MN then sends *CN_address* messages that contain the IPv6 addresses of the CN with which the MN usually communicates. The SN then forwards the messages to the GW.

When the *join_request* message reaches the GW, it creates a binding entry for the MN and, assigns MN with a unique 16 bits ID. Also the GW maps the IPv6 address of its CNs to a unique 16 bits address. This is done in order to save the number of bits used in the IP addressing, which might waste useful energy and bandwidth of the SNs when routing the data and mobility-related packets of the MNs. Then the GW sends a *join_confirm* message to the SN that forwards it to the MN. The *Join_confirm* message contains MN’s ID and the short address of its CNs.

If, in future, the MN wants to communicate with a CN for the first time then the MN will request the GW to provide a 16 bits address by sending CN’s alias name. Thus the GW will run an Address Resolution Protocol (ARP) and provides a 16-bits address against the CN’s IP address or its alias name. Similarly, when a packet for the MN can arrive from CN that is located outside the PAN, for which the GW does not have a pre-existing binding. In this case, the GW will first create a binding and sends this binding to the MN before sending the data packet.

### 5.2. Handoff Support

**Figure 3.**
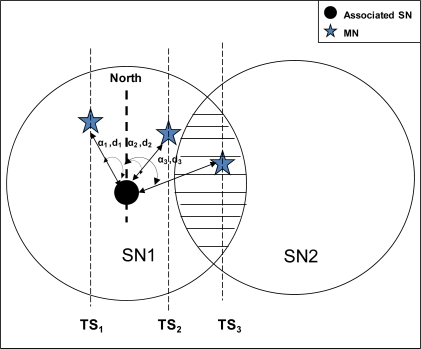
Next node activation process using AoA method.

The proposed handoff support for MN considers the sleep state of SNs. Thus, as shown in Figure 3, once the current SN observes that its link quality with the MN has degraded beyond a certain threshold value, it then concludes that the MN is moving. Hence the current SN needs to activate the next appropriate SN for the handoff process. In order to activate the next appropriate SN, the associated SN must know the direction in which the MN is moving. The direction can only be found through localization procedure, where a series of MN’s locations depict its course of movement, i.e., tracking. There are various methods to obtain MN location like, trilateration/triangulation method, AoA (Angle of Arrival), and ToA / TDoA (Time {Difference} of Arrival) method, signal strength indicator etc.

In order to determine the direction of movement, we propose to use AoA method [21]. AoA is defined as the angle between the propagation direction of an incident wave and some reference direction, which is known as orientation. Orientation, defined as a fixed direction against which the AoAs are measured, is represented in degrees in a clockwise direction from the North. When the orientation is 0° or pointing to the North, the AoA is absolute otherwise it is relative.

As shown in Figure 3, ‘the star’ shows MN position at time-stamps TS_1_, TS_2_, and TS_3_ respectively. The associated SN measures the AoA from the signal that it receives from the MN. Moreover, the associated SN also estimates the distance between the MN and itself by using received signal strength of the packets.

As shown in Figure 3, let α_1_, d_1_ ; α_2_, d_2_ ; α_3_, d_3_ be the angles (in degrees) and the distances between the SN1 and the MN, at time-stamps TS_1_, TS_2_ and TS_3_ respectively. Based on the angle and distance information, the SN can obtain the MN’s location coordinates. At the time-stamp TS_3_, the SN1 awakes the next SN2 for the handoff. Also SN1 sends a *new_node* message to SN2 that contains MN’s ID.

**Figure 4.**
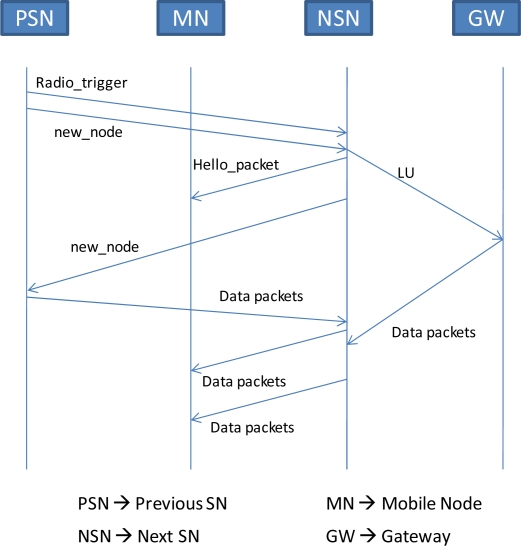
MN’s handoff support scenario.

When the next SN receives the *new_node* message, it transmits a *hello_packet* at some intervals. Also, at the same time, it sends an LU to the GW. When the next SN detects the MN, it again sends a *new_node* message to the previous SN. The purpose of this message is to create a tunnel between the next SN and the previous SN, so that as soon as the previous SN receives the *new_node* message, it starts forwarding the MN’s packets to the new location. The tunnel’s outer header contains the next SN as a destination and previous SN as a source. When the MN’s packets stop arriving at the previous SN, the SN switches to the sleep mode after a short interval of time. The handoff support mechanism is depicted in Figure 4. If the next SN does not detect the MN within a certain amount of time, it will consider that the MN is lost. So the next SN will request, by sending a radio triggered broadcast, to all of its neighboring SNs to suspend their duty cycle and keep awake until the MN is found.

### 5.3. Proposed Message Formats

This section introduces some of the mobility-related signaling message formats needed to support a MN within the PAN. The message formats make use of 6LoWPAN’s adaptation layer’s reserved dispatch values, in order to differentiate between different kinds of mobility messages exchanged between SNs and GW [20]. Every adaptation layer packet begins with a dispatch value that identifies the type of header to be followed next. The dispatch value, an 8 bits value, indicates what kind of packet is it. For example, a dispatch value of 01 000001 indicates that the following header is an uncompressed IPv6 header. The pattern 01 000010 represents that the following header is fully compressed from 2 bytes to 40 bytes. When there are more than one LoWPAN headers, they should appear in the following order: Mesh header, Fragmentation header, and Header Compression header. Also the dispatch headers appear before each header [20]. Moreover, 16 bits addressing scheme is used in order to route the data packets to and from a MN. The proposed message formats which utilize the adaptation layer reserved dispatch values and 16 bits addressing scheme are given below.

#### 5.3.1. Location Update Message Format

**Figure 6.**
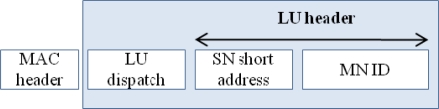
Location update (LU) message format.

Figure 6 shows the format of an LU message that a SN sends to the GW when the MN associates with it.

The SN sends an LU message with a unique dispatch. The dispatch indicates that the message is an LU message that has to be forwarded to the GW. The message also contains the 16 bits short address of the source SN. The intermediate nodes relay the LU packet to the GW by checking the dispatch value. When the GW receives the packet it identifies the packet as LU message and updates the entry of the MN by replacing the previous SN’s short address with the next SN’s address. The entry for the MN remains in the binding table of the GW, as long as the GW keeps receiving LU messages on behalf of MNs. When the GW stops receiving the LU messages of MNs, it deletes its entry in the binding table after the timeout of which the exact value is implementation specific.

#### 5.3.2. Message Format of a Data Packet Sent from a MN to the CNs

**Figure 7.**
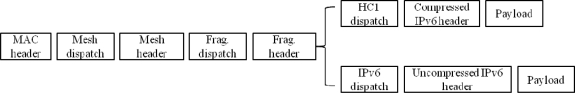
A typical 6lowpan message format of a data packet.

The MN’s data packet format is shown in Figure 7. It should be noted that the Fragmentation header is optional and is used when the packet size is large enough to be fit into the Maximum Transmission Unit (MTU) for IPv6 packets over IEEE 802.15.4. HC1 is the Dispatch header that indicates a compressed IPv6 header. An uncompressed IPv6 header is represented by IPv6 dispatch.

**Figure 8.**
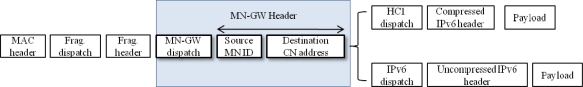
Message format of a data packet sent from the MN to the CN via the tunnel from MN’s associated SN to the GW.

When the SN receives the data packet, it first checks whether it has a routing entry for the destination or not. If the destination CN is located within the PAN, the SN forwards the packet to the node closer to the CN. If the SN is unable to locate the destination within the PAN, then it forwards the packet to the GW. In this case, it takes off the mesh header and inserts MN-GW dispatch and the MN-GW header. MN-GW dispatch indicates to the forwarding nodes that the source of the packet is a MN, and the packet needs to be forwarded to the GW. The packet’s header includes source MN’s address and destination CN’s address. Thus using the dispatch value eliminates the need of having a tunnel between the SN and the GW. The packet format of the MN’s data packet that the SN forwards to the GW is shown in Figure 8.

When the GW receives the packet, it takes off the MN-GW header, assembles the fragments, and decompresses the IPv6 header, if needed before forwarding the packet to CN.

#### 5.3.3. Message Format of a Data Packet Sent from a CN to the MN

**Figure 9.**
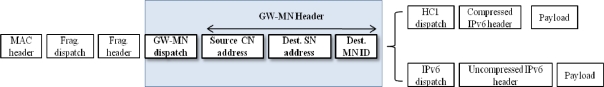
Message format of a data packet sent from the CN the MN via the tunnel from GW to SN.

When a CN, which is located either outside or inside the PAN, wants to send a data packet to a MN; it first sends the packet to the GW. This is because the GW has a binding cache which stores the mapping of MN’s ID with its current location in terms of SN’s short address. The GW then fragments and compresses the received packet, if needed. Thereafter, it attaches GW-MN dispatch and the packet’s header. The dispatch indicates that this packet is for the MN and sent by the GW. The packet’s header contains 16 short bits address of the SN, with which the MN is currently associated, the source CN’s 16 bits address, and the destination MN’s 16 bits ID. The message format is shown in Figure 9.

The packet, sent by the GW, is then relayed over a number of hops as it reaches the destination SN. By looking at the dispatch value, the intermediate relaying nodes know that the packet is sent from the GW. When the destination SN receives the packet, it takes off GW-MN dispatch and the destination SN’s address from the packet; and forwards it to the MN. Thus in this mobility support scheme we propose to identify mobility-related packets with the use of specific dispatch values. The dispatch values of some of the messages are shown in Table 1 below.

## 6. Distributed LoWMob (DLoWMob) Mobility Support Scheme

In this section, we propose the DLoWMob scheme, a distributed version of LoWMob. DLoWMob enhances the performance of LoWMob by introducing the concept of MSP. It reduces the signaling traffic at the GWs and enables the route optimization in 6LoWPAN. While LoWMob enables 6LoWPAN mobility without having MNs involved in most of the mobility-related signaling, it causes the GWs to be involved in all of the mobility-related signaling and data packet handling. That is, whenever a MN associates with a new SN, the SN has to send an LU messages to the GW that could negatively affect the lifetime of the SNs and ultimately the whole PAN. Moreover, the MN’s data packets are routed through the GW; since it has the binding information of MN’s ID with its current associated SN, thereby increasing the traffic concentration at the GW.

**Figure 10.**
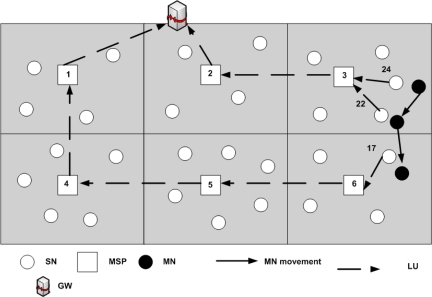
Updating binding information with MSPs.

In DLoWMob, the PAN is assumed to be partitioned into multiple regions, as shown in Figure 10. Each region has one MSP and its associated SNs which are managed by the MSP. Hence there exists a hierarchy in the PAN. The GW manages MSPs only, while each MSP manages its associated SNs in its region.

The main functions of the MSP are two-fold; firstly, it limits the number of LUs to the GW by managing the binding information between MNs and SNs in its region locally. That is, it reports LUs to the GW only when a MN enters in its own region. While a MN moves in its region, from a SN to another SN in the region, no LU is required to be sent to the GW. Consequently, MSPs could reduce significant network bandwidth and the energy consumption. Secondly, it enables the data packets between MNs and its CNs in a PAN to be routed between their corresponding MSPs. That is, it removes the need of having every data packets passing through the GW, thus making route optimization possible. The next two subsections describe each function in detail.

### 6.1. The Handoff Process in DLoWMob

The Handoff process in DLoWMob is basically the same as LoWMob, except for the MSP’s participation. When a MN moves closer towards the next SN, the previous SN notifies the next SN about the handoff by sending a *new_node* message. When receiving the *new_node* message, the next SN sends an LU to the MSP. The MSP checks whether it has an entry for the MN or not. If the MN’s previous SN and next SN are registered with the MSP, i.e., the both SNs are in the same region of the MSP, the MSP updates its binding table by modifying the entry of the previous SN. In this case, it does not forward the LU to the GW. Otherwise, it sends the LU to the GW, so that the GW can update its binding table for the MN. The binding information in the MSP for the MN remains as long as there is an active communication between the MN and its CN. If the MN does not send or receive data packets within MSP, the MSPs can delete the MN’s entry because the MN is assumed to be dead, as explained in the System Model section.

Figure 10 shows a MN which is moving from SN24 to SN17 via SN22 within a PAN. When a MN enters the coverage area of an MSP for the first time, then the MSP sends an LU to the GW with the help of other MSPs. For example, when a MN is associated with SN24 (i.e., it enters into the region of MSP3), MSP3 sends an LU to the GW. When the MN is associated with SN22, MSP3 does not send an LU to the GW because the movement is within intra-region, and the binding information is managed by the MSP itself.

### 6.2. Route Optimization for Intra-PAN Communication in DLoWMob

We propose a Route Optimization scheme for an Intra-PAN communication in order to reduce the traffic concentration at the GW. This could extend the lifetime of the nodes near the GW, thereby extending the network lifetime also. We also propose a handoff mechanism for Route Optimization scheme. The mechanism could support the handoff for the communication even when the source and destination are mobile. The next two subsections describe routing of data packets between source and destination MN and the handoff process in more details.

#### 6.2.1. Routing of MN’s Packet in a Route Optimized Environment

The MSPs in DLoWMob enables the route optimization between multiple corresponding MNs by creating and storing the routing entries, specific to the MNs in their routing table. Based on the routing table entries, the proposed mobility header facilitates routing between a source and the destination MSP.

The routing of MN’s packets with the help of MSPs has two stages. In the first stage, a source MSP tries to locate a destination MN’s MSP on behalf of the source MN. In this process, source and destination MN’s specific routing entries are created in the MSPs that fall within the path of the source and destination MSP. In the second stage, the data packets are routed based on the routing entries created in the MSPs and the proposed mobility header and dispatch.

**Figure 11.**
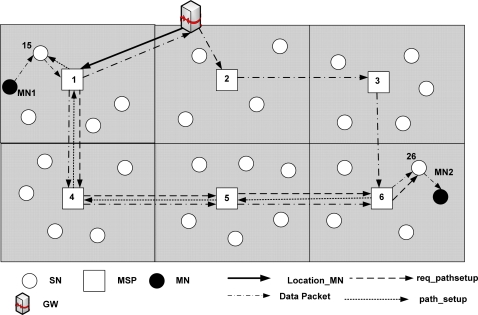
DLoWMob mobility scheme with Route Optimization.

Figure 11 shows two MNs, as located within the same PAN. MN1 is associated with SN15, whereas MN2 is associated with SN26. When MN1 wants to send a data packet to MN2 for the first time, it first sends it to SN15. The packet format can be the same as described in Figure 7.

When SN15 receives a data packet from MN1, it forwards the packet to MSP1. MSP1 checks whether it has an entry for the MN2 in its routing table or not. If the source MSP, i.e., MSP1 does not find a routing entry for the destination MN, it takes off the mesh header from the data packet and adds MN-GW dispatch and MN-GW header to the data packet. The source MSP then forwards the data packet to the GW, which in turn advances it to the destination MSP using the packet format as shown in Figure 9. However note that in Figure 9, destination SN address will be replaced by destination MSP address. When the packet reaches the destination MSP, the destination MSP replaces its address with SN’s address with which the MN is associated.

**Figure 12.**
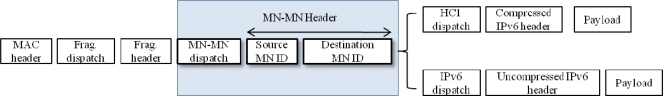
Message format of a data packet from source MN to destination MN via MSPs.

Also at the same time, the GW sends a *location_MN* message to the source MSP. The *location_MN* message contains the address of the destination MSP. Once the source MSP receives the location information of the MN, it sends a *request_pathsetup* message to the destination MSP, which contains the IDs of both MN1 and MN2. When the destination MSP, i.e., MSP6, receives *request_pathsetup* message, then it forwards the message to the SN with which the destination MN is associated, i.e., SN26. The destination MSP, i.e., MSP6, then sends a *path_setup* message back to the MSP1 that contains MN2 and MN1’s ID. When forwarding the *path_setup* message back to MSP1, the MSPs en route records destination and source MSP address, IDs of both MNs, and the MSP from which it has received a *path_setup* message. Thus each MSP would get a routing table entry in order to route the packets between the MNs. When MSP1 receives *path_setup* message, it creates a routing entry for the destination MN, i.e., MN2; and forwards *path_setup* message to SN15 with which MN is currently associated. Moreover, when the *path_setup* message reaches the source MSP, it immediately stops sending the MN’s data packet to the GW. Instead, it attaches MN-MN header and dispatch to the data packets and forwards the packets to the MSP from which it received the *path_setup* message. The packet format is shown in Figure 12.

For instance, in Figure 11, the MSP1 forwards MN1’s data packet to MSP4. MSP4 confirms, by checking the MN-MN dispatch, that it is a data packet towards a MN. Thereafter, it verifies whether it has any record in its routing table about the possible current location of the destination MN, (MN2 in Figure 11). Since it has already created a record from the *path_setup* message, it forwards the packet to the next MSP on the route (i.e., MSP5 in Figure 11). This process continues until the packet reaches the destination MSP (i.e., MSP6 in Figure 11). The destination MSP then forwards the data packets to the destination SN (i.e., SN26 in Figure 11) using the packet format of Figure 9. However, in this case the dispatch and the header will be MSP-SN.

#### 6.2.2. Handoff Process in the Route Optimization Scheme

In the route optimized scheme when a MN moves to a different MSP while communicating with another MN, a new path is needed to be established between the destination and source MSP. This section introduces the modified version of the handoff process (which is explained in section 5.2) to enable creation of a new path between source and destination MSP.

**Figure 13.**
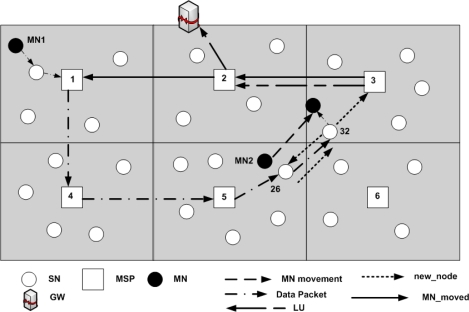
Handoff process in DLoWMob with Route Optimization scheme.

In Figure 13, when the communication link between the MN2 and SN26 degrades, SN26 sends a *new_node* message to the next or future SN i.e., SN32. This time *new_node* message contains MN1’s ID and its MSP’s short address, i.e., MSP1’s address, along with MN2’ID. Then SN32 forwards the *new_node* message to its MSP, i.e., MSP3. MSP3 then sends an LU to the GW, and *MN_moved* message to MSP1. The *MN_moved* message contains the addresses of both of the MSPs, as source and destination, and the MN’s addresses. The MSPs, which forward the *MN_moved* message to the MSP1, record the two MN’s IDs and their respective MSPs. The purpose of this message is to create route specific entries on behalf of the two MNs. When the message reaches MSP1, it broadcast the message to its one hop neighbors (MSPs).This is done so that in case if MN1 has also moved to a new MSP, then the new MSP is informed about the destination MN’s movement. Moreover, once the *MN_moved* message reaches MSP1, it immediately starts forwarding the MN1’s data packets, if any; to the next MSP from which it received the *MN_moved* message i.e., MSP2. When MSP2 checks the destination of the packet, it knows that the MN2 is associated with MSP3 and forwards the packet to the next MSP, i.e., MSP3 (which is the destination MSP as well). Meanwhile, until the *MN_moved* message reaches the MSP1, MSP1 keeps sending the packets to the old path. The packets are delivered to the MN2 through the tunnel created between the current SN and the previous SN.

Similarly, when the source MN, i.e., MN1, moves while sending the data packets to MN2, its current SN will inform the next SN about its corresponding MN’s MSP. Also the current SN will notify the next SN about its own MSP so that the next MSP can forward MN’s data packet to the previous MSP since it already has a path towards the destination MN. Meanwhile, the next MSP can send a *MN_moved* to the destination MN’s MSP, in order to establish a path between itself and destination MSP. It should also be noted that the routing table entries of MSPs will expire when the source stops sending data packets to destination MN.

## 7. Security Considerations for Proposed Mobility Schemes

This section discusses the security model of the proposed mobility schemes in this paper. The following assumptions and notations are used in order to describe the security processes and cryptographic operations in this paper:

1. In order to establish an individual key shared between the GW and a node, and a pairwise key shared between a node and each of its intermediate one hop neighbors, we can use LEAP [22]. In the proposed scheme, the MSPs share an individual key with the GW and pairwise key with each of its intermediate MSP. The SNs also share pairwise key with another SN and an individual key with the GW. It should be noted that both individual key and pairwise key are symmetric keys.
2. G: The gateway (GW) which is assumed to be trusted [23].
3. MSP_i_, MSP_j_: Two intermediate neighboring MSPs located in a path between a source MN and a destination MN.
4. K_ij_: A symmetric pair-wise shared key between MSP_i_ and MSP_j_
5. K_s_: Session key shared between source and destination MN.
6. K_ig_: Individual shared key between MSP*i* and the GW.
7. K_m_: Mobile Key between MN and its future SNs
8. K_c_: Communication key which is shared between a MN and the GW.
9. M1|M2: The concatenation of messages M1 and M2
10. {M}_<Kij ,C>_: The encryption of message M, with key K_ij_ and the incremental counter C [24].
11. K_MH_: The key shared between MN and its HA.
12. K_SP_: The key shared between SN and its MSP.
13. K_SG_: The key shared between SN and GW.
14. MAC (K_ij_, M): The message authentication code (MAC) of message M, with the symmetric secret key K_ij_

### 7.1. Authentication of LU

An attacker can pretend to be an MSP and can send an LU on behalf of the MN. This attack can be avoided if the LU is authenticated using the MAC value and by encrypting the message by the symmetric key shared between the source MSP and the GW, i.e., K_ig_. The following message format can be used when sending an LU to the GW [25]:

$${\text{MSP}}_{i}\to G:{\left\{\text{LU}\right\}}_{<\mathrm{Kig}>},{\text{MAC}\{K}_{\text{ig}},C|{\left\{\text{LU}\right\}}_{<\mathrm{Kig}>}\}$$

When the GW, receives the LU it verifies (1) whether the request is originated from the claimed sender by checking MAC and K*_ig_*, and (2) whether the message is replayed by a malicious MSP by checking counter value C in the MAC. If any verification is not found correct, then the message is dropped by the GW.

### 7.2. Authentication of the MN before It Is Allowed to Join the PAN

When a new MN enters a PAN for the first time, it informs a SN about its own home address and HA’s (Home Agent’s) address. Also the MN generates a random number (R1), known as challenge, and encrypts it with the shared key between itself and it’s HA, i.e., K_MH_. Then the MN sends this encrypted message to its associated SN. The SN forwards the message to its MSP by encrypting it with the key shared between itself and GW, i.e., K_SG_. The MSP then forwards the message to the GW. When the GW receives the message, it decrypts it with the key K_SG_ and gets to know that this message is sent by the legitimate SN and the message is destined for MN’s HA. The GW then informs the MN’s HA that the MN is currently located in its PAN. Following this, a security association is set up between the GW and HA, using IKE [26]. It should be noted that the legitimacy of the HA can be established during the creation of security association between GW and HA, using IKE. This is because IKE uses public key and certificates signed by a trusted authority [27]. Thereafter, the GW forwards the MN’s challenge to the HA, using the newly created security association. The HA then decrypts the number (R1) using K_MH_ and generates a random number (R2) and encrypts the concatenate of R1 and R2 with MN’s and its shared key, i.e., K_MH_. The HA then sends the encrypted concatenation of R1 and R2 to GW. When this information reaches the GW, the GW forwards the information to the MSP by encrypting it with K_SG_. The MSP then forwards the message to the SN. The SN decrypts with K_SG_, and then delivers the message to the MN. Once the MN receives this information, it decrypts the message with K_MH_. The MN then verifies whether it has received R1 or not in the message. The MN then reverses the concatenation of R1 and R2 and encrypts the concatenation with the K_MH_ and forwards the message to its SN. The SN encrypts the message with K_SG_ and sends to the MSP that then forwards to the GW, as described above. When the GW receives this message, it forwards the message to the MN’s HA after decrypting with K_SG_. The HA then verifies the MN’s authenticity by checking whether it received R2 or not. If the HA is satisfied with the authenticity of the MN, it sends a *MN_authorized* message to the GW, which indicates that MN is a legitimate node. Moreover, the HA update the binding table entry of the MN with its current CoA. On the other end, GW assigns two keys to the MN. The first one is known as Mobile Key (K_m_) and the second key is known as Communication Key (K_c_). The GW then sends these keys to the HA. Also it sends K_m_ to the SN by encrypting them with K_SG_. In order to securely deliver the Mobile Key and Communication Key to the MN, the HA encrypts the K_m_ and K_c_ with K_MH_ and forwards the encrypted keys to the GW. The GW then forwards this message to SN by encrypting it with K_SG_. The SN in turn sends it to the MN. The MN decrypts the message and obtains the K_C_ and K_m_.

### 7.3. Mutual Authentication of MN and its Future SNs

**Figure 15.**
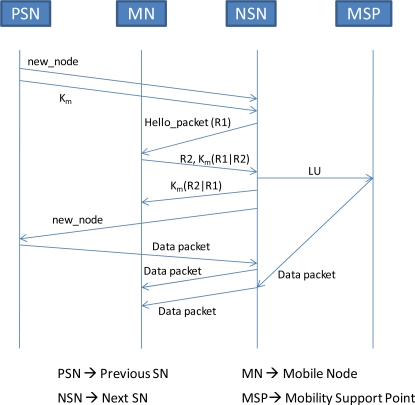
Secure Handoff.

When the MN moves within the PAN, it associates with different SNs in the PAN. In order to provide mutual authentication between the MN and its next SN, the current SN informs the next SN, during handoff, about K_m_. The current SN achieves this by encrypting the K_m_ with the pairwise key shared between itself and the next SN. The mutual authentication process using K_m_ is shown in Figure 15.

In the hello packets, the next SN includes a random number R1. When MN receives this number, it generates another random number R2 and sends R2 to next SN along with the concatenation of R1 with R2 after encrypting the concatenation with K_m_. Once the next SN verifies that the sender knows the K_m_, it sends an LU to MSP by encrypting with the shared key K_SP_. Also it reverses the order of the concatenation of R1 and R2 and encrypts the concatenation with K_m_ before sending it to MN.

Note that as the MN moves on then the Mobile Key, i.e., K_m_, would be exposed to many of the SNs within the PAN. Thus it is desirable that the GW revoke the key after some time. In order to do that, the GW generates K_m_ after some amount of time. The time interval at which the key is revoked is implementation specific. The GW then sends this key to the MN by encrypting it with K_c_. At the same time, the GW sends the key to the current SN by encrypting it with the key shared between it and the SN.

### 7.4. Securing MN’s Data Packets

When the MN first time sends a data packet to its CN, it encrypts the data packet with K_c_ and forwards it to its SN. The SN then forwards it to its MSP, which in turn forwards MN’s packet to the GW. The GW then decrypts the message by K_c_ of the source MN and forwards the data packet to the destination MSP, using the Communication Key (K_c_) of the destination MN. Also, at the same time, the GW creates a session key, i.e., K_s_, for the communication between source MN and destination MN. Next the GW informs the source MSP about the destination MSP’s address. Also it sends the session key to source and destination MNs by encrypting the session key with their respective K_c_. When the source MN sends a data packet to destination MN, the data packet can be encrypted using the session key as shown below [25]:

$${\text{MN}}_{\text{source}}\to {\text{MN}}_{\text{destination}}:{\left\{\text{data}\right\}}_{<\mathrm{Ks}>},{\text{MAC}\{K}_{s},C|{\left\{\text{data}\right\}}_{<\mathrm{Ks}>}\}$$

Hence using K_s_, both MNs can communicate securely.

### 7.5. Authentication of Messages that Modify a MN’s Routing Entry in the MSPs

In the proposed scheme, signaling messages, such as *MN_moved* and *path_setup* messages, create or modify the routing state of a MN in an MSP and establish a path between source and destination MSPs. Thus it is necessary to ensure that these signaling messages are sent by an authentic source. Thus whenever an MSP sends the signaling message (that modifies or creates the routing state) to the next hop MSP; in the path between source and destination MSPs it encrypts the message with the pair-wise key of itself and its next hop MSPs. The message format is shown below:

$${\text{MSP}}_{i}\to {\text{MSP}}_{j}:{\left\{\mathrm{MN}\_\mathrm{moved}\right\}}_{<\mathrm{Kij}>},\;\text{MAC}\{K_{\text{ij}},\;C|{\left\{\mathrm{MN}\_\mathrm{moved}\right\}}_{<\mathrm{Kij}>}\}$$

When the next MSP receives the packet, it establishes the authenticity of the message by verifying whether or not it has received the messaged from a trusted MSP by computing the MAC using the pairwise key. Also it creates or updates the routing state of the MNs. It then forwards it to its next hop MSP, towards the destination MSP, by encrypting the message with a pairwise key shared between itself and the next hop MSP. This process enables creation or alteration of routing state entries of MNs securely.

## 8. Performance Evaluation

We have evaluated the proposed LoWMob scheme by developing a complete simulation in Qualnet v4.5 and through numerical analysis. The terrain area is 2,500 × 2,500 m^2^. A total of 50 SNs are deployed in an 8 × 8 logical grid. The main reason of dividing the whole area into a grid is to examine the MN behavior at each step. We have used the random way point mobility model and the fluid flow mobility model. The minimum speed of MN is 1 m/s, and the maximum speed varies to 20 m/s, 25 m/s, 30 m/s, and 35 m/s. The MN pause time is 30 sec. AODV is used as a routing protocol. The simulation is run for 500 seconds and there are 20 simulations run.

The performance metrics of interest are (a) End-to-End delay: delay of packets transmitted from MN to the GW, and the number of packets that are transmitted from one MN to the other MN; (b) packet success ratio: the number of packets successfully received by the GW, out of the ones that are transmitted by a MN; and for the communication between multiple MNs packet success rate is the number of packets that are successfully received by a MN out of the ones that are transmitted by another MN (c) hand off overhead.

### 8.1. End to End Delay and Packet Success Ratio

**Figure 16.**
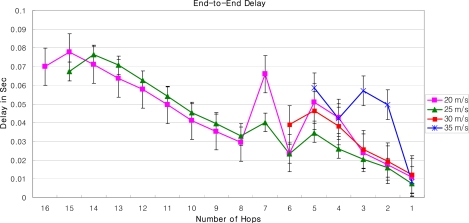
Delay vs. number of hops with variable MN’s speed (LoWMob).

**Figure 17.**
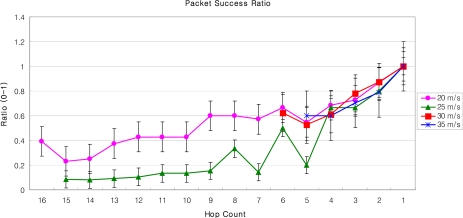
Packet success rate vs. number of hops with variable MN’s speed (LoWMob).

Figure 16 and 17 describe the end-to-end delay and the packet success ratio of the packets from MNs to the GW when the speed of MNs and the number of hops between them varies. After a certain number of hops, the end-to-end delay increases linearly with the increasing number of hops between the MN and the GW. Also the end-to-end delay increases when the speed of the MN increases. This is because as the speed of the MN increases, the association of the MN with its SN breaks, triggering off the handoff process. Thus when the MN moves with high speed, most of the time is spent to complete the handoff process by the new SN and the old SN.

Some spikes in the graph can also be observed for some early hops. There are several factors which cause these spikes. These include: i) the mobility model (the random way point) – because the MN abruptly changes its position according to the mobility model used [28], ii) the speed of MN – that causes handoff and some nodes might not have routing information, iii) The pause time between movements, and [29] iv) AODV – AODV broadcasts packets bring traffic in the network that not only cause collision but also introduce hidden node problem [28]. The issues could be fixed with different network topologies, different speed, and pause time of the MN; and with the use of static routing etc.

The packet success ratio, when the MN is far away from the GW, i.e., 16 hops, is just about 0.4 for a MN moving with the speed of 20 m/s. As the number of hops between the GW and the MN decreases packet success ratio increases. As the MN reaches closer the GW the success ratio approaches to 1, and the end-to-end delay to 0.01 seconds. Moreover, it can be seen from Figure 17 that when the speed of the MN is 20 m/s the packet success ratio is better than when the speed is 25 m/s. This is because as speed increases the number of handoff increases, which can lead to a significant packet loss. Also, when the speed increases exponentially, there is a possibility that the MN will be lost in the PAN. This is because, as the new SN wakes up for the handoff process, the MN may have already crossed the new SN. As shown in Figure 17, when the MN is 5 hop away from the GW, the packet success ratio at the speed of 30 m/s is almost double than that of speed of 25 m/s. It should be noted that this type of anomaly in the graph is observed due to i) the speed of the MN, that triggers routing path discovery more frequently, ii) the link congestion, and iii) the duty cycle of SNs. Note that the link congestion is not caused by the SNs which are relaying packets, but the SNs around the MN which are participating in broadcast messages for AODV. Also note that we have used standard MAC Protocol of IEEE 802.15.4, i.e., CSMA/CA, as underlying MAC protocol. The results of the packet success ratio, and the end-to-end delay could be improved significantly for MAC Protocols, like X-MAC [30], C-MAC [31] or RI-MAC [32].

The performance of our LoWMob scheme in terms of end to end delay and packet success ratio is good when the MN is closer to the GW. But usually it is not the case, because the MN can roam anywhere within the network. Moreover, if the network size increases the performance of our LoWMob scheme decreases dramatically. Also as the speed increases the number of handoff increases, thus degrading the network lifetime.

**Figure 18.**
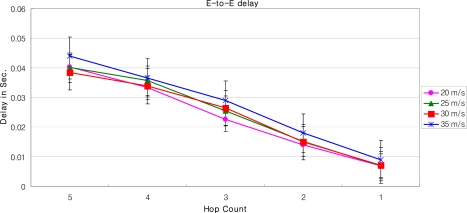
Delay vs. number of hops with variable MN’s speed (DLoWMob).

**Figure 19.**
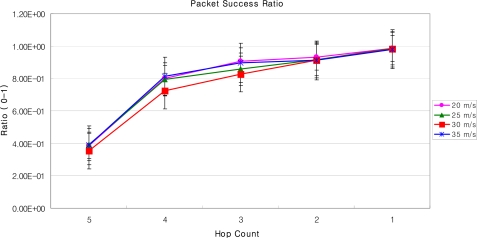
Packet success rate vs. number of hops with variable MN’s speed (DLoWMob).

In order to simulate DLoWMob in Qualnet v. 4.5, we have deployed MSPs (In our simulation, PAN coordinator is represented as MSP) in such a fashion that they could find the shortest path towards the GW. The results shown below depict the scenarios when MN’s current MSP sends a MNs packet to the GW or to another MSP. As shown in Figure 18 and Figure 19, at any speed of the MN, the data packets are always bridged over five hops. It is observed that, at any time instant, the MN finds itself within the vicinity of MSP and there is a fix cost from MSP to the other MSP, or to the GW. Moreover, unlike the case of our LoWMob scheme, the end to end delay and packet success ratio do not change significantly even at different speeds of the MN. Even though, when the MN is moving in a high speed and incurring frequent handoffs, the number of hops between the MSP and the GW are reduced. The end to end delay for the DLoWMob scheme has been reduced up to twice of the delay as recorded in the LoWMob scheme. Similarly the packet success ratio is observed to be 0.79, i.e 79%, as compared to 0.4, 40%, as observed in the LoWMob scheme. This because the number of hops in the DLoWMob is less as compared to the original scheme. This decreases the delay and increases the packet success ratio in DLoWMob.

**Figure 20.**
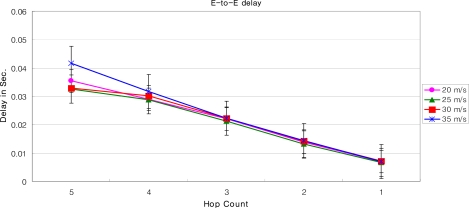
Delay vs. number hops with variable MN’s speed (DLoWMob with Route Optimization).

**Figure 21.**
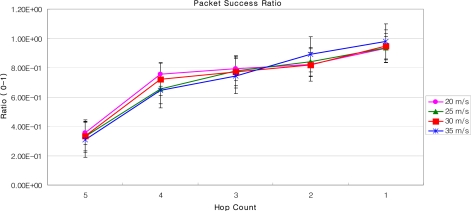
Packet Success Rate vs. number of hops with variable MN’s speed (DLoWMob with Route Optimization).

Figures 20 and 21 show the end-to-end delay and the packet success ratio for DLoWMob with Route Optimization, respectively. In this case, both MNs send packets to each other while following random way point mobility model. It can be seen that the performance of our DLoWMob scheme with single MN and with multiple MNs is almost similar. At any speed and at any location within the network, both MNs are separated at most by a five hop count, and it is the matter of fact that with the lesser number of hops, the packet’s reliability and QoS increases. High throughput is achieved by DLoWMob when MN’s target MSPs communicate directly; this limits the number of hops between any source and any destination within the network. Also, as long as the source and the destination MNs do not change their MSPs, there is no need to make new paths between the source and the destination MSPs. This reduces the number of messages needed to establish the paths.

### 8.2. Handoff Overhead

**Figure 22.**
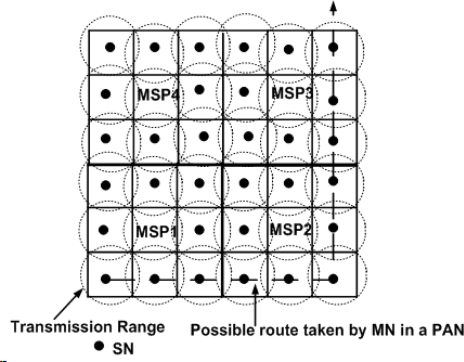
Association Region of SNs in the network topology.

The handoff overhead is the signaling overhead that is incurred when the MN breaks off its association with its previous SN and attaches with a next SN. This includes the signaling cost for the handoff, like sending *new_node* and LU messages. In order to derive the handoff overhead through analytical modeling, we consider Figure 22. The circular dotted line is the transmission range of the SNs, whereas the SNs are organized in a grid. The squares in the Figure represent the Association Range (AR) of a SN. In other words, when the MN is within the square then it is associated with one particular SN. When it moves out of the square, the signal strength between the MN and its current SN decreases and it associates with the next SN. Also, there are 4 MSPs located in the PAN. In order to derive the handoff signaling overhead, we determine the average number of SNs with which a MN associates, when fluid flow mobility model is used.

For evaluation purposes, we assume the following:

1. A domain is composed of *N* equal squares that are equal in size and form a contiguous area.
2. The MNs are moving with an average speed of *v*, and the direction of their movement is between *0* to *2π*.
3. MN moves out of the PAN within *K* finite movements

In [33] the authors proved that *μ(k)* is the average number of SNs with which a MN associates with, and it is rarely sensitive to change of *K*, unless *K* is much smaller than *N*. Thus we assume *K* equals *N*.

The signaling cost in the LoWMob scheme can be broken down to:

1. The cost incurred in sending LU messages to the GW, and the cost of sending *new_node* messages when a MN associates with a new SN.
2. The cost incurred in tunneling data packets from the GW to the SNs, for the destination MN. The default parameter values for calculation of LU and tunneling cost are given in Table 2.

In Table 2, the LU message cost for one hop is 48 bits. Moreover, in order to deliver the data packet to the MN, extra 16 bits are appended that represent the SN’s short address.

#### 8.2.1. Location Update Cost

Let LU_LoWMob_ be the LU cost, in bits, incurred due to MN’s K movement inside a PAN for LoWMob:

(1) $${\mathrm{LU}}_{\mathrm{LoWMob}}=\pi_{0}\times S_{\mathrm{LU}}+(\mu (K_{1})-1)\times S_{\mathrm{LU}}+(\mu (K_{1})-1)\times S_{\mathrm{NM}}\times 2$$

(2) $$S_{\mathrm{LU}}=b\times L_{1}$$

(3) $$\pi_{0}=\frac{1}{2}$$

Similarly for DLoWMob, the LU cost can be given by:

(4) $${\text{LU}}_{\text{DLoWMob}}=\pi_{0}\times S_{1}+(\mu (K_{2})-1)\times S_{2}+(\mu (K_{1})-1)\times S_{\mathrm{NM}}\times 2+(\mu (K_{1})-1)\times S_{3}$$

(5) $$S_{1}=b\times L_{1}\;(\mathrm{number of hops from SN to GW})$$

(6) $$S_{2}=b\times L_{2}\;(\mathrm{number of hops from MSP to GW})$$

(7) $$S_{3}=b\times L_{3}\;(\mathrm{number of hops from SN to MSP})$$

Equation 1 includes both the LU cost to the GW and the cost to send *new_node* message. Also *π_0_* is the equilibrium probability of the state *0* that represents the probability of MN staying outside a given PAN. In this case, we assume that the probability of MN staying outside the PAN is same as staying inside. For the LoWMob scheme *μ(K_1_)* is calculated as 2.8, from the equations derived in [33]. Also it can be seen from [33] that *μ(K_1_)* is not affected by the speed. For DLoWMob, *μ(K_2_)* is calculated as 0.999969 and is less than *μ(K_1_)* as MSPs area of coverage is larger than a single SN. Thus the MN stays within an MSP’s region for a longer amount of time; this reduces the number of LUs to the GW. Also the average number of hops between a MSP and GW is less as compared to the number of hops between SN and GW.

#### 8.2.2. Tunneling Cost

Equation 8 gives the tunneling cost required to tunnel MN’s packet from the GW:

(8) $${\mathrm{TU}}_{\mathrm{cost}}=\lambda \times T_{\mathrm{as}}\times \mu (k)\times S_{T}$$

(9) $$S_{T}=t\times L$$

In the above equation, *λ* is the average packet arrival rate of a MN, when it is associated with the SN. *L* is the average number of hops the packet is tunneled from GW to SN. The time for which a MN remains connected to a SN can be referred to as *T_as_* [33]:

(10) $$T_{\mathrm{as}}=\frac{\surd (\pi A)}{2v}$$

where, *A* is the area of the square, shown in Figure 22.

##### 8.2.2.1. Location Update Signaling Cost

**Figure 23.**
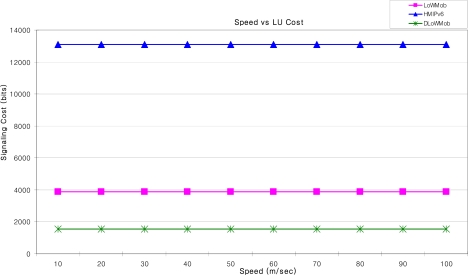
Speed vs. LU cost.

The dotted line in Figure 22 shows a possible path followed by a MN when it enters and exits a PAN. Figure 23 shows the LU cost (in bits) incurred due to the MN’s movements along the same path, at different speed. It shows that as the speed increases, LU cost does not change. This is due to the fact that even if the speed of the MN increases, it associates with the same number of SNs that lay in its path [34]; and thus these SNs send the same number of LU messages. Also, the signaling cost of DLoWMob to send an LU message is less than that of LoWMob. This is because for DLoWMob, an LU message travels less number of hops to reach the GW. Also the MSPs in DLoWMob limit the frequency of sending LUs to GW.

Moreover, it can be seen that the signaling overhead of the proposed schemes is significantly less as compared with HMIPv6. This is because the adaptation layer packets have been used for sending LU, instead of IP packets (as in the case of HMIPv6). In HMIPv6 the size of the LU message is 68 bytes [33].

##### 8.2.2.2. Tunneling Cost versus Packet Arrival Rate

**Figure 24.**
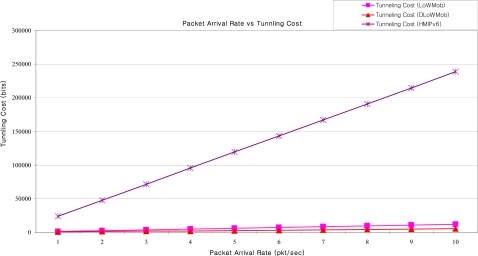
Tunneling cost vs. packet arrival rate.

From Figure 24, it can be seen that the tunneling cost of both DLoWMob and LoWMob scheme increases linearly as the packet arrival rate increases. This is because the tunneling cost is directly proportional to the packet arrival rate. However, the increase in the tunneling cost for the LoWMob scheme is more, as compared to DLoWMob; because, comparably with DLoWMob, LoWMob involves more number of hops from GW to the MN. The comparison with the proposed scheme also shows that the tunneling cost is less as compared to HMIPv6. This is because in HMIPv6, tunneling cost for a packet is equal to the size of an IPv6 header [33].

## 9. Conclusions

This paper discussed a network-assisted mobility support scheme for 6LoWPAN nodes, which are extremely energy and resource constrained devices in nature. The paper proposed the LoWMob mobility support scheme, where a MN associates with the SN, which is responsible for mobility of the MN. The proposed scheme reduces the tunneling overhead between the GW and the SN, without involving MN in a significant amount of signaling. Signaling overhead has been reduced at a greater extent by introducing a special packet format that utilizes the dispatch values of the 6LoWPAN adaptation layer and 16 bits addressing of IEEE 802.15.4. Furthermore, this paper proposed a distributed version of LoWMob, named as DLoWMob. It employs the concept of MSPs and route optimization for intra-PAN communication. Mobility-related packets are handled by the MSPs that route them to and from the CN or the GW. We have shown that at different speed of the MN, DLoWMob shows better performance than LoWMob in terms of end-to-end delay, packet success ratio, and signaling costs.

## Figures and Tables

**Figure 5.**
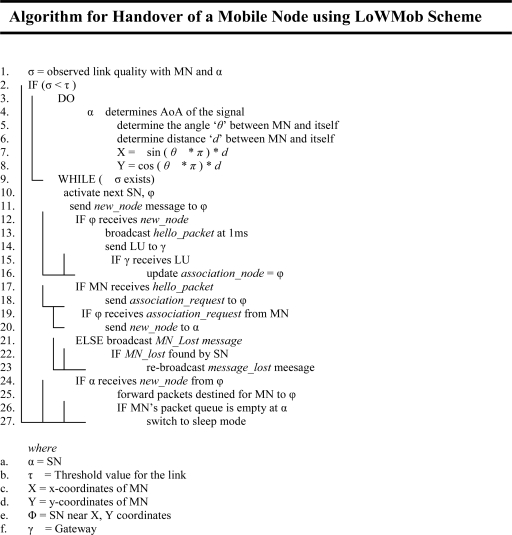
The algorithm for the handoff support.

**Figure 14.**
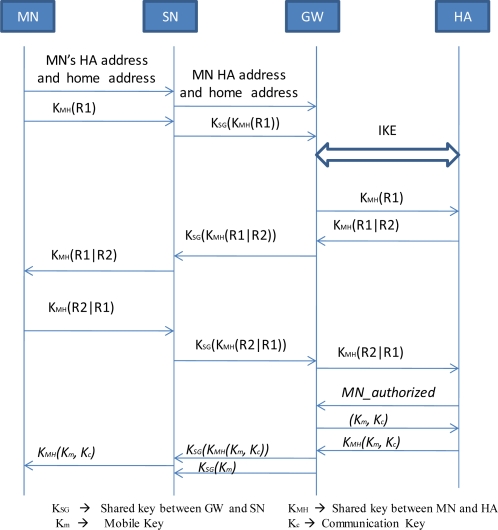
Authentication of a new MN.

**Table 1.** Dispatch values for the message.

**Dispatch value**	**Message types**
01 000111	Join_request
01 001011	Association_request
01 001001	New_node
01 000110	GW-MN (Data packet from GW to MN)
01 001111	MN-GW (Data packet from MN’s associated SN to GW)
01 000111	LU (Location update packet)
01 010001	MN-MN (Data packet from source MN to destination MN)

**Table 2.** The default parameter values for calculation of LU and tunneling cost.

**Name**	**Description**	**Value**

b	LU message cost for one hop	144 bits
t	Tunneling cost in one hop	16 bits
S_NW_	new_node message cost in one hop	160 bits
N_SN_	Number of SNs	36
N_MSP_	PAN size in terms of number of MSPs	4
S_AR_	Area of the Association Range	900 m^2^
SD	Area covered by an MSP	8,100 m^2^
v	Average speed of MN	10 m/s
μ(K_1_)	Average number of SNs a MN associates with in a PAN	2.8
μ(K_2_)	Average number of MSP a MN associates with in a PAN	0.999969
L_1_	Average number of link hops between GW and SN	10
L_2_	Average number of link hops between GW and MSP	5
L_3_	Average number of link hops between MSP and SN	1
